# Assessing gastro-intestinal related quality of life in cystic fibrosis: Validation of PedsQL GI in children and their parents

**DOI:** 10.1371/journal.pone.0225004

**Published:** 2019-12-20

**Authors:** Mieke Boon, Ine Claes, Trudy Havermans, Victoria Fornés-Ferrer, Joaquim Calvo-Lerma, Inês Asseiceira, Anna Bulfamante, María Garriga, Etna Masip, Sandra Woodcock, Sylvia Walet, Celeste Barreto, Carla Colombo, Paula Crespo, Els Van der Wiel, Jessie Hulst, Sandra Martinez-Barona, Rita Nobili, Luisa Pereira, Mar Ruperto, Saioa Vicente, Kris De Boeck, Carmen Ribes-Koninckx, for the MyCyFAPP consortium

**Affiliations:** 1 Cystic Fibrosis Center, Department of Pediatrics, University Hospital Gasthuisberg, Leuven, Belgium; 2 Instituto de Investigación Sanitaria La Fe de Valencia, Spain; 3 Associação para a Investigação e Desenvolvimento da Faculdade de Medicina, Lisbon, Portugal; 4 Università degli Studi di Milano, Fondazione IRCCS Ca’ Granda, Ospedale Maggiore Policlinico, Milan, Italy; 5 Hospital Universitario Ramón y Cajal, Madrid, Spain; 6 Erasmus Medical Center, Sophia Children’s Hospital, Rotterdam, Netherlands; Fordham University, UNITED STATES

## Abstract

**Background:**

Most patients with cystic fibrosis (CF) suffer from pancreatic insufficiency, leading to fat malabsorption, malnutrition and abdominal discomfort. Until recently, no specific tool was available for assessing gastro-intestinal related quality of life (GI QOL) in patients with CF. As the Horizon2020 project MyCyFAPP aims to improve GI QOL by using a newly designed mobile application, a sensitive and reliable outcome measure was needed. We aimed to study the applicability of the existing child-specific Pediatric Quality of Life Inventory, Gastrointestinal Symptoms Scales and Module (PedsQL GI) in children with CF.

**Methods:**

A multicenter, prospective observational study was performed in 6 European centers to validate the PedsQL GI in children with CF during 3 months.

**Results:**

In total, 248 children and their parents were included. Within-patient variability of PedsQL GI was low (24.11), and there was reasonable agreement between children and parents (ICC 0.681). Nine of 14 subscales were informative (no ceiling effect). The PedsQL GI and the median scores for 4 subscales were significantly lower in patients compared to healthy controls. Positive associations were found between PedsQL GI and age (OR = 1.044, p = 0.004) and between PedsQL GI and BMI z-score (OR = 1.127, p = 0.036). PedsQL GI correlated with most CFQ-R subscales (r 0.268 to 0.623) and with a Visual Analogue Scale (r = 0.20).

**Conclusions:**

PedsQL GI is a valid and applicable instrument to assess GI QOL in children with CF. Future research efforts should examine the responsiveness of the CF PedsQL GI to change in the context of clinical interventions and trials.

## Introduction

Cystic Fibrosis (CF) is the most common life-threatening autosomal inherited disease in Europe where currently over 44,000 cases of CF are registered [1]. It is a multisystemic disorder with main involvement of the respiratory and gastro-intestinal tract. Pancreatic insufficiency (PI) leads to steatorrhea, abdominal distension and/or discomfort, flatulence, constipation and poor weight gain. Treatment consists of pancreatic enzyme replacement therapy (PERT), high caloric diet and supplementation with fat soluble vitamins [2–3]. In CF, nutritional status has a major impact on the progression of pulmonary disease [4]. Particular attention is needed to prevent an imbalance between energy losses (due to PI), increased energy needs (due to pulmonary disease) and usual energy intake [2,5].

Despite treatment with PERT, close nutritional follow-up and multidisciplinary support, residual fat malabsorption occurs in a considerable number of patients with CF [6] resulting in nutritional deficiencies [7] and abdominal symptoms [8]. Up to now, there was hardly any evidence about the optimal dose of PERT needed nor how to adapt this to individual patient’s needs [2,9].

The MyCyFAPP project aims to develop and validate a mobile application (APP) to optimize and personalize the intake of PERT [10]. An algorithm, integrated in the APP, was developed to determine the optimal PERT dose needed per meal, based on *in vitro* data of food digestion [11–12]. In the final phase of the project, a European multicentre prospective clinical trial was planned to assess the impact of the use of the APP on patient outcomes. Quality of life (QOL) assessment as a Patient Reported Outcome (PRO) is a widely accepted clinical endpoint in clinical trials [13–14]. Quality of life (QOL) is an overarching term, reflecting the expectation of an individual of the quality of various domains in life. Quality of life has been widely studied, mostly using self-reports. As QOL is a very relevant outcome from a patient-perspective, gastro-intestinal related QOL (GI QOL) was chosen as the primary outcome for the MyCyFAPP trial.

A literature search resulted in the identification of two relevant existing QOL questionnaires. The first is the CF-specific QOL questionnaire, the CFQ-R [15–17], to be used for children and their parents from 6 years onwards. Thus far, only the respiratory domain of this scale has been approved as endpoint by the FDA [12] and was used as a primary outcome parameter in only a few clinical trials [18–19]. The child version of CFQ-R includes a domain assessing abdominal quality of life (digestion), but this scale includes only 7 items and does not cover all relevant GI aspects in CF; it was therefore considered not suitable as a primary outcome parameter in the MyCyFAPP project. Very recently, the abdominal domain was expanded for the adults and teen versions only [20], so it is not applicable for the current project.

The second scale is the Pediatric Quality of Life Inventory, Gastrointestinal Symptoms Scales and Module (PedsQL GI) [21]. It is a generic symptom measurement instrument, consisting of 14 GI related subscales. It is feasible in (young) children with abdominal symptoms, can discriminate between health and disease and has been validated in patients with several gastrointestinal diseases (i.e. Crohn’s disease, gastroesophageal reflux disease and functional gastro-intestinal disorders) [22–23]. Previously, it was shown that the subscales have a high internal consistency [22]. The PedsQL GI, however, has not been validated for use in children with CF yet.

Because of its proven usability in chronic GI illnesses, the PedsQL GI was selected as a candidate measure to be used in the MyCyFAPP trial. The present study aims to investigate the psychometric characteristics of the PedsQL GI and assess the usability of the PedsQL GI in children with CF. The aim was to study the natural variability of the PedsQL GI subscales, the relation to validated QOL scales (CFQ-R, Visual Analogue Scale (VAS)), ceiling and floor effects, agreement between children and parents, correlation with clinical data (e.g. age, BMI,…) and finally identify the most informative subscales to be used in the MyCyFAPP project. Therefore, a multicenter, prospective observational study was performed with evaluation of PedsQL GI, CFQ-R and VAS at 2 time points with a clinically stable interval of 3 months.

## Materials and methods

### Study population

A 3-month observational, multicenter study was performed in stable children with CF 24 months to 18 years of age, all having PI (stool elastase < 200 mcg/g stool) and treated with PERT. A three-month follow up time period was chosen as convenience for the patients, as this would coincide with regular outpatient clinic.

The children were recruited in the 6 European CF centers participating in the MyCyFAPP project (Lisbon, Madrid, Milan, Leuven, Rotterdam, Valencia). The diagnosis of CF had been confirmed by a sweat chloride concentration ≥ 60 mEq/L and/or the presence of 2 disease-causing mutations in the *CFTR* gene. Patients and their parents were informed about the study during the outpatient clinic.

As the majority of children in each center would be included in the trial, a control group could not be established. The results from healthy controls were used from published data in a US population [23].

Exclusion criteria were: an acute infection associated with decreased appetite or fever, acute abdominal pain necessitating an intervention at the time of inclusion, physical findings that would compromise the safety of the participant or the quality of the study data as determined by the investigators, investigational drug use within 30 days prior to inclusion or recent start on CFTR modulator therapy within 3 months prior to inclusion.

The study was approved by the ethics committees of all 6 participating centers (University Hospital Gasthuisberg, Leuven; Instituto de Investigación Sanitaria La Fe, Valencia; Ospedale Maggiore Policlinico, Milan; Hospital Universitario Ramón y Cajal, Madrid; Erasmus Medical Center, Sophia Children’s Hospital, Rotterdam). Parents signed an informed consent form for their own participation and for the participation of their child; in accordance with local regulations, adolescents aged 12 years and older signed an assent or consent form.

### Questionnaires

Patients filled out the age-appropriate versions of the PedsQL GI [24], CFQ-R [16] and a Visual Analogue Scale (VAS) at baseline (M0) and after 3 months (M3) during regular outpatient clinics. Parents and children were asked to fill out the questionnaires independently.

The PedsQL GI is a symptom-specific questionnaire to measure gastrointestinal symptoms in children 2–18 years [21–23]. It consists of 74 questions in 14 subdomains (including stomach pain, constipation, worries about bowel movement,…). The feasibility is high (easy to fill out with only 1,69% missing for children, 1,84% missing for adults) and the estimated completion time is 15 minutes. Normal values are available. The subscales have a high internal consistency (reliability of all subscores > 0,70 and >0,90 for total score) and discriminate between health and disease. The subscales can be used individually and for each subscale the minimal clinical significant change is known. A higher score indicates better QOL. PedsQL GI is abnormal in several disorders (Crohn’s disease, constipation,…).

The CFQ-R evaluates general quality of life in patients with cystic fibrosis from the age of 6 years in several subdomains (including physical functioning, treatment burden, respiratory symptoms,…). The subscales have a high internal consistency, discriminate between health and disease and a good construct validity was shown [16].

The VAS consists of a linear scale to answer the question ‘How is your health state today?’ from 0 to 100%. It is an easy and fast to use tool that is well validated and has an added value on the evaluation of generic QOL.

A parent report was included for all age groups for the PedsQL GI and the VAS, and for the CFQ-R for children aged 6–14. For children < 4 years old, only a parent report for the PedsQL and VAS was included. The CFQ-R has child versions for children older than 6 years. See S1 Table for an overview of the combination of the different scales used. The same versions were used at both study visits, independently from changing age category.

In preparation of this study, we paid the rights to use the PedsQL GI, in consultation with Dr. Varni, the intellectual owner of the PedsQL GI. As contractually required, and following previous research using PedsQL scales, the PedsQL GI was translated forward and backward into Dutch, Flemish, Italian, Portuguese and Spanish, and the translations were validated by interviewing a minimum of 5 test patients/parents per version to assess comprehensibility, relevance as well as construct and content validity of the translations. This procedure was in accordance with the instructions from Dr. Varni.

A 3 or 5-point response scale is used for scoring of all individual items of the PedsQL GI and it’s subscores. Items are reverse-scored and linearly transformed to a 0–100 scale so that 100% corresponds to no symptoms and 0% to the presence of severe symptoms in that domain.

### Clinical data

Spirometry, height, weight and BMI were assessed at M0 and M3. Spirometry data were calculated according to the GLI references [25], height, weight and BMI according to the CDC references [26]. The daily dose of PERT (LU/kg/day) used was registered at M0 and M3.

The study started in October 2016 and finished in May 2017.

### Statistical analysis

#### Patient characteristics

Clinical data were summarized by median scores (1st, 3rd Q) in the case of continuous variables and by relative and absolute frequencies in the case of categorical variables.

Data on PedsQL GI in patients with CF were compared to those published for healthy controls, using linear regression [23]. Factor analyses could not be performed due to the small sample size of participants in each age group. Cronbach alpha was used to determine internal consistency of scales and subscales: scales with a value >0.7 are recommended for comparing patient groups whereas those with a value >0.90 are recommended for comparison within individuals [27].

The anticipated differences (effect sizes) between patients with CF and healthy controls were estimated by taking the difference between the healthy sample means and the CF sample means, divided by the pooled SD (Cohen’s D). Effect sizes for differences in means are designated as small (0.20), medium (0.50), and large (0.80). The minimal important difference (MID) was calculated as the standard error of the measurement (SEM) divided by the square root of 1-Cronbach alpha [27].

#### PedsQL GI natural variability

Intraclass Correlation Coefficient (ICC) and confidence intervals (CI) were calculated to assess the reliability between patients (between patient’s variability) and parents, as well as between months (within patient variability). Taking into account the ratio between subject variability and total variability, the ICC provides an index of absolute agreement by means of one-way ANOVA. ICC values below 0.5, between 0.5 and 0.75, between 0.75 and 0.9, and greater than 0.90 are indicative of poor, moderate, good, and excellent reliability, respectively [28].

#### Association between PedsQL GI and patient characteristics

Beta mixed regression was performed to study the association between PedsQL GI and several covariates that were selected according to the expert knowledge: Age, BMI z-score, FEV_1_ z-score, gender, dose of PERT and time (change between first and second evaluation).

To select subscales of importance for use in the clinical trial, we used the feedback from the interviews of patients/parents (see above) and we set a 95% cut-off: subscales with a median value >95% demonstrated a ceiling effect and are thus unlikely to be informative to study an intervention.

#### Correlation between PedsQL GI and CFQ-R, VAS

The Spearman correlation coefficient was calculated between PedsQL GI and each CFQ-R's subscale score. The same analyses were performed with the CF PedsQL GI.

The validation process included data collection at baseline and 3-months. Pooled data were used to calculate the correlation between PedsQL GI and CFQ-R as well as the correlation between PedsQL GI and VAS, both for patients as for parents.

### CF PedsQL GI

The shortened, CF specific version of PedsQL GI (CF PedsQL GI) was defined as the mean score of all informative subscores i.e. those with a median result ≤95%.

All analyses were performed using R software (version 3.4.1.) and packages ICC (version 2.3.0), MBESS (version 4.3.0), glmmADMB (version 0.8.3.3) and clickR (0.4.04).

## Results

### Patient characteristics

Two hundred and forty-eight children were included in the study at M0 (Table 1), 239 completed the second questionnaire at M3. At least one parent was included per child. Forty-eight children were between 2 and 4 years old and thus could not fill in a child version of the questionnaire themselves, so 200 patient questionnaires were collected at M0.

**Table 1:** Patient characteristics.

Variable	n = 248 Mean (SD)
Age (y)	9.21 (5.3, 13.58)
PERT intake (LU/day)	187 500 (105 000, 280 000)
PERT intake (LU/kg/day)	6 233.97 (3 926.11, 9 465.22)
Weight (CDC z-score)	-0.23 (-0.91, 0.26)
Height (CDC z-score) BMI (CDC z-score)	-0.12 (-0.86, 0.49) -0.29 (-0.92, 0.27)
FVC z-score	-0.49 (-1.48, 0.45)
FEV_1_ z-score	-1.02 (-2.3, 0.16)
FEV_1_/FVC z-score	-0.95 (-1.82, -0.26)
FEF_25-75_ z-score	-1.19 (-2.57, -0.06)
Girl (n, %)	116 (46.8%)
**CF center** (n, %)	
Lisbon	42 (16.9%)
Rotterdam	29 (11.7%)
Valencia	41 (16.5%)
Leuven	42 (16.9%)
Madrid	32 (12.9%)
Milan	62 (25%)

Abbreviations: y = year, LU = lipase units, CDC = Center for Disease Control, BMI = body mass index, FVC = forced vital capacity, FEV_1_ = forced expiratory volume at 1 second, FEF_25-75_ = forced expiratory flow between 25–75% of the pulmonary volumeThe median total child-reported PedsQL GI at baseline was 85.5 (IQR 78.6–92.6) and was significantly lower than in healthy controls (median 89.8, p 0.04) (Table 2) [23]. Median values were significantly lower compared to healthy controls for 4 subscales: ‘Diarrhoea’, ‘Constipation’, ‘Gas and Bloating’ and ‘Worry about stomach aches’. For one subscale, ‘Blood in bowel movement’, patients with CF had higher (better) values than healthy controls. Similar results were obtained from parents (S2 Table). The internal consistency was good for most subscales. Data for age subgroups are presented in S3 Table and S4 Table and show only small differences due to lower patient numbers.

**Table 2:** Results for the total PedsQL GI in children with CF, obtained in our study at baseline, compared to published healthy controls [23].

	Healthy Controls	Patients with CF n = 248	Cronbach α	MID **	Linear Regression	Effect Sizes
	Median (1st, 3rd Q.)	Median (1st, 3rd Q.)			Difference [CI 95%]; p-value	
**Total PedsQL GI (%)**	89.8 (79.14, 98.41)	85.5 (78.55, 92.6)	0.94	2.33	-2.15 [-4.22, -0.08] p = 0.04*	0.215
**Stomach_Pain (%)**	81.6 (67.04, 93.95)	83.3 (73.95, 95.8)	0.84	7.42	0.91 [-2.24, 4.07] p = 0.57	-0.05
**Stomach_Discomfort (%)**	93.29 (79.68, 100)	95 (85, 100)	0.72	8.15	1.89 [-0.79, 4.56] p = 0.16	-0.125
Food_Drink_Limits (%)	94.14 (80.09, 100)	95.8 (83.3, 100)	0.80	7.33	2.63 [-0.19, 5.46] p = 0.07	-0.16
Trouble_Swallowing (%)	97.28 (89.26, 100)	100 (91.7, 100)	0.49	8.35	1.78 [-0.09, 3.65] p = 0.06	-0.15
**Heartburn_reflux (%)**	92.49 (81, 100)	87.5 (75, 93.8)	0.52	9.37	0.5 [-1.77, 2.77] p = 0.66	-0.04
Nausea_vomiting (%)	92.49 (81, 100)	100 (81.2, 100)	0.79	7.32	2.09 [-0.52, 4.70] p = 0.12	-0.13
**Gas_and_bloating (%)**	85.57 (70.47, 98.73)	75 (64.3, 89.3)	0.81	8.65	-9.36 [-12.76, -5.95] p < 0.001*	0.47
**Constipation (%)**	90.09 (74.88, 100)	85.7 (73.2, 94.6)	0.89	4.78	-3.92 [-6.56, -1.27] p = 0.004*	0.26
Blood bowel movement (%)	98.15 (88.24, 100)	100 (100, 100)	0.64	4.47	4.74 [3.21, 6.28] p < 0.001*	-0.54
**Diarrhea (%)**	97.35 (87.52, 100)	85.7 (78.6, 100)	0.76	6.37	-4.68 [-6.79, -2.57] p < 0.001*	0.34
Worry_bowel_movements (%)	97.79 (85.1, 100)	100 (85, 100)	0.73	7.59	-0.49 [-2.81, 1.82] p = 0.67	0.03
**Worry_stomach_aches (%)**	92.89 (80.18, 100)	87.5 (62.5, 100)	0.73	11.25	-5.19 [-8.51, -1.87] p = 0.002*	0.25
**Medicines (%)**		87.5 (68.8, 93.8)	0.55	11.84		
**Communication (%)**		90 (65, 100)	0.79	10.54		

Note that no data were available in literature for the subscales ‘Medicines’ and ‘Communication’. Subscales with a median score ≤95% in patients with CF are marked in bold. Significant differences between patients with CF and healthy controls are marked with *.

Abbreviations: MID minimal important difference

The subscales without ceiling effect are: ‘Stomach pain and hurt’, ‘Stomach discomfort when eating’, ‘Heartburn and Reflux’, ‘Gas and Bloating’, ‘Constipation’, ‘Diarrhoea’, ‘Worry about Stomach Aches’, ‘Medicines’, and ‘Communication’ (Table 2). These subscales are all clinically relevant for patients with CF and were thus included in the ‘CF PedsQL GI’. In the parents’ questionnaires, the same subscales had mean values ≤95% (S2 Table).

### Natural variability

The ICC between M0 and M3 measurement was moderate to good for most of the subscales, which implies reasonable reproducibility (Table 3). ICCs between children and their parents were also moderate for most subscales, indicating that both children and their parents perceive the child’s symptom profiles similarly. The within-patient variability (24.11) was much lower than the between-patients variability (77.75).

**Table 3:** Intraclass correlation coefficients for the total score and subscales of the PedsQL GI.

Subscale	*Intraclass Correlation Coefficient Between Measurements (M0-M3) Children*			*Intraclass Correlation Coefficient Between Measurement (M0-M3) Parents*			*Intraclass Correlation Coefficient Between Children and Parents Measurements (M0)*			*Intraclass Correlation Coefficient Between Children and Parents Measurements (M3)*		
	*ICC [CI 95%]*			*ICC [CI 95%]*			*ICC [CI 95%]*			*ICC [CI 95%]*		
**PedsQL GI Total Score**	**0.764**	[0.698,	0.829]	**0.768**	[0.711,	0.826]	**0.681**	[0.599,	0.763]	0.671	[0.589,	0.754]
**Stomach Pain and Hurt**	0.59	[0.495,	0.686]	0.671	[0.599,	0.743]	0.538	[0.439,	0.637]	0.665	[0.585,	0.746]
**Stomach Discomfort When Eating**	0.611	[0.519,	0.703]	0.694	[0.626,	0.761]	0.566	[0.472,	0.66]	0.627	[0.539,	0.714]
**Food and Drink Limits**	0.57	[0.47,	0.669]	0.558	[0.467,	0.648]	0.597	[0.507,	0.687]	0.399	[0.276,	0.522]
**Trouble Swallowing**	0.486	[0.374,	0.599]	0.66	[0.586,	0.733]	0.351	[0.228,	0.474]	0.43	[0.311,	0.549]
**Heartburn and Reflux**	0.529	[0.424,	0.635]	0.663	[0.59,	0.736]	0.527	[0.427,	0.628]	0.528	[0.424,	0.632]
**Nausea and Vomiting**	0.608	[0.515,	0.701]	0.664	[0.592,	0.737]	0.645	[0.564,	0.725]	0.62	[0.531,	0.709]
**Gas and Bloating**	0.665	[0.583,	0.748]	0.68	[0.609,	0.75]	0.614	[0.527,	0.701]	0.545	[0.442,	0.647]
**Constipation**	0.71	[0.636,	0.783]	0.761	[0.705,	0.817]	0.646	[0.563,	0.728]	0.563	[0.462,	0.663]
**Blood bowel movement**	0.143	[-0.005,	0.292]	0.469	[0.367,	0.571]	0.424	[0.309,	0.539]	0.169	[0.022,	0.316]
**Diarrhea**	0.646	[0.56,	0.731]	0.7	[0.633,	0.766]	0.491	[0.385,	0.597]	0.57	[0.473,	0.668]
**Worry about bowel movements**	0.754	[0.692,	0.817]	0.726	[0.665,	0.788]	0.554	[0.458,	0.651]	0.401	[0.277,	0.524]
**Worry about Stomach Aches**	0.47	[0.355,	0.585]	0.568	[0.48,	0.657]	0.341	[0.216,	0.466]	0.482	[0.37,	0.593]
**Medicines**	0.692	[0.616,	0.769]	0.721	[0.658,	0.783]	0.584	[0.493,	0.676]	0.482	[0.37,	0.594]
**Communication**	0.7	[0.626,	0.775]	0.658	[0.584	0.733]	0.291	[0.16,	0.422]	0.409	[0.287,	0.531]

### Association between PedsQL GI and patient characteristics

**Fig 1:**
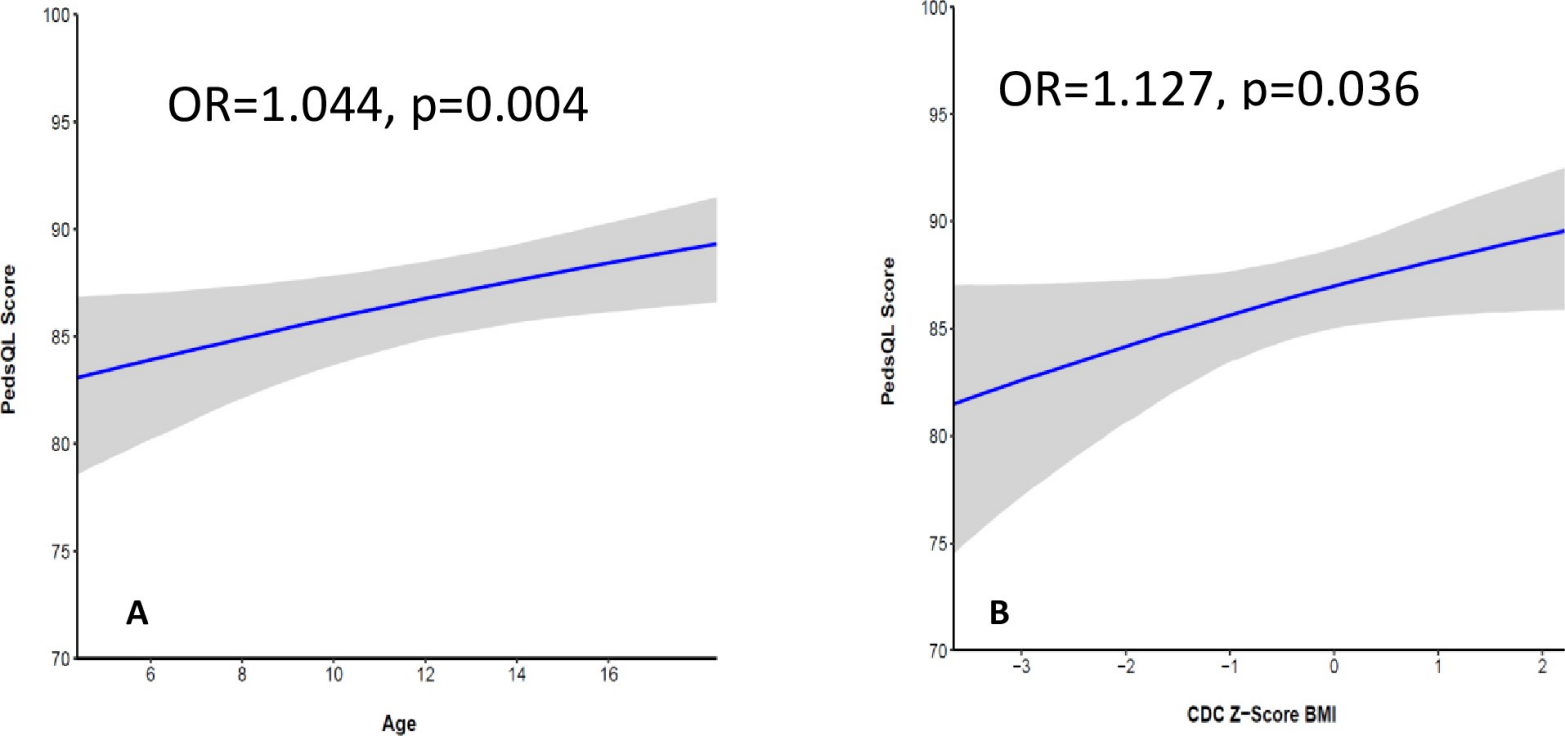
**Association between PedsQL GI and age (1A) and BMI (1B).** Abbreviation: OR = Odd’s ratio.

Beta mixed regression was performed to study the association between PedsQL GI and several covariates: age, BMI z-score, FEV_1_ z-score, gender and time (first or second visit) (Table 4). Because FEV_1_ and BMI were not independent, FEV_1_ was discarded from the model. There was a positive association between PedsQL GI and BMI z-score (OR 1,127, 95% CI 1,008–1,261, p = 0.036) as well as age (OR 1,044, 95% CI 1,014–1,076, p = 0.004). (Fig 1). There was no association between PedsQL GI and gender, time and FEV_1_ z-score. Using height z-score instead of BMI z-score, we didn’t detect a significant difference (OR 1.002, 95% CI 0.916–1.096, p = 0.97). There was no association between PedsQL GI and total PERT dose (OR 0.877, 95% CI 0.754–1.019, p = 0.086).

**Table 4:** Results of the Regression model to assess the association of patient characteristics on PedsQL GI.

	Estimate	Odd’s ratio	CI 95%		P-value
(Intercept)	3.008	20.247	[3.319	123.531]	0.001
Age	0.043	1.044	[1.014	1.076]	**0.004**
Z-score BMI	0.12	1.127	[1.008	1.261]	**0.036**
Gender—girl	-0.158	0.854	[0.691	1.054]	0.141
Time study–m+3	0.039	1.04	[0.944	1.145]	0.43
log(pert_intake_m1)	-0.132	0.877	[0.754	1.019]	0.086

### Correlation between PedsQL GI and CFQ-R, VAS

There was a significant, but weak correlation between PedsQL GI and VAS (rho = 0.20, p<0.001). Most of the subscales of CFQ-R correlated weakly, but significantly with PedsQL GI for each patient age group (S5 Table).

### CF PedsQL GI

Table 5 demonstrates the results for the CF PedsQL GI for patients and controls and their parents’ scores. The CF PedsQL GI correlated highly with the total PedsQL GI (r = 0.93, p < 0.001) (S1 Fig).

**Table 5:** Results for the CF PedsQL GI.

CF PedsQL GI	Median (1st, 3rd Q.) (%)	Cronbach’s alpha	Wilcoxon rank sum test
Patients with CF	83.30 (75, 91.20)	0.931	p < 0.001
Healthy Controls	87.66 (82.10, 91.66)		
Parents of children with CF	82.65 (75.60, 90.30)	0.943	p < 0.001
Parents of healthy Controls	89.55 (85.21, 93.35)		

Internal consistency of the CF PEDsQL, and most subscales of the CF PedsQL GI was high (Tables 2 and 5).

## Discussion

We studied the psychometric properties, the validity and applicability of the existing PedsQL GI Module as measurement instrument for use in children with CF and their parents.

We confirm that GI QOL scores, assessed by PedsQL GI, are lower in the studied CF population (both patients and parents) compared to (published) scores of healthy controls [23]. We found low values (≤95%) for 9 of the 14 subscales of the PedsQL GI, with most reported symptoms in the subscales “gas and bloating”, “stomach pain”, “constipation” and “diarrhea”. This confirms clinical observations in children with CF [8–9]. The PedsQL GI subscales are GI symptom specific and detailed. This makes the PedsQL GI suitable for use in CF. In addition, scores on the subscales “worry about medicines” and “worry about communication” were low. This information is relevant for the final phase of the MyCyFAPP project, in which the impact of the use of a newly designed APP on GI QOL will be studied [10].

These results point to the important psychological burden of the GI symptoms in children with CF and affirms concerns from patients and parents; when questioned about their priorities for research in a systematic survey (James Lind Alliance project), patients put treatment burden and GI symptoms very high on the list [29].

A ceiling effect was observed for 5/14 PedsQL GI subscales. This corresponds with the clinical infrequency or absence of symptoms assessed by these subscales in patients with CF. For example, the subscale ‘blood in bowel movement’ had a mean value of 100%, indicating no problems at all. This corresponds with the clinical experience that bloody stools are extremely rare in children with CF. Theoretically they can occur after bleeds from esophageal varices in patients with the complication of liver cirrhosis and portal hypertension. However, in this context hematemesis or melena is more common. In addition, systematic follow up of liver disease and if detected, proactive treatment will prevent this complication in most patients.

The intraclass correlation between scores from patients and their parents was acceptable for the total score and for most subscale scores. This suggests that children and their parents agree in their perception of the symptoms and that their paired data could be compared across age groups in clinical research.

There was no change in GI QOL during the short study period indicating an excellent test-retest reliability. The three-month period (regular out-patient clinics) may be considered long to examine test-retest reliability, but this period was chosen not to overburden patients. Cronbach alpha for the total PedsQL GI and the CF PedsQL GI was >0.90, confirming that the scales can be used for comparison within and between individuals. There was more variability within individual age subgroups because of lower patient numbers. However, the PedsQL GI was designed for use among age groups with the possibility of working with all versions together in the MyCyFAPP project.

A significant association between PedsQL GI and BMI was found. In children with CF, an improved BMI generally reflects a better nutritional status and a correlation was expected with higher self-reported QOL. This finding supports the construct validity of the questionnaire and the possibility to create a CF version.

Construct validity for most subscales of the PedsQL GI was shown by the association with established QOL measures, the CFQ-R subscales and VAS. These correlations were significant, but weak, as anticipated, because the questionnaires assess different domains of QOL. As expected, the strongest correlation was seen between PedsQL GI and the CFQ-R domain ‘digestion’.

In this study, we found a weak, but significant positive association between PedsQL GI and age. This suggests that patients become less symptomatic when they grow older or ‘get used’ to their GI symptoms. This finding demonstrates the clinical importance of care of gastro-intestinal symptoms in younger children with CF.

For the first time, an existing GI-specific QOL questionnaire was examined for use in CF. This is of high clinical and research value. Most patients with CF report abdominal symptoms, which could not be monitored in detail until now, as the existing CFQ-R, a questionnaire on general QOL in patients with CF, only contains 7 GI related items. Further study of the CF PedsQL GI will assess its value as a more specific and sensitive outcome to assess GI QOL, useful both in clinical settings and for research purposes: for example, trials to assess the efficacy of CFTR modulator therapies. To date, only BMI and weight are used as nutritional outcomes in clinical trials, but these do not reflect abdominal discomfort or abdominal symptoms.

Very recently, the JenAbdomen-CF score was developed [8]. This newly constructed questionnaire is also very detailed and aims to represent CF-specific abdominal symptoms. Reports show correlations with findings on abdominal ultrasound [30], significant discriminative validity and low natural variability in patients older than 6 [31]. However, there are no data on comparison with existing questionnaires and data are scarce for the age group <6. In addition, the potential for use as a self-report measure for children has not yet been established, which is an important asset of the CF PedsQL GI.

We obtained repeated observational data of a large group of patients from several centers and countries and their parents, so the data are robust. In addition, the translation to 5 different languages is an asset.

A limitation of the study is that no study specific control group was included. The control group used consists of North-American children without gastro-intestinal disease [21]; they are slightly older and have a different ethnic background. For this control group, internet-based questionnaires were used in contrast to paper forms filled out during the outpatient clinic visit for our patients. There are no data on BMI, weight and height for the control subjects. As BMI was a contributing factor in the CF group, this might induce bias. However, since we plan to use the CF PedsQL GI for interventional studies in CF, this is of minor importance as in the MyCyFAPP trial patients will be used as their own control. Although beyond the scope of the present study, a limitation may also be the fact that known-group differences were not investigated (f.e. discrimination between patients with and without history of DIOS).

The interviews with patients during the detailed translation process showed that the items of the PedsQL GI are in line with the GI symptoms patients with CF experience most frequently. A limitation is that we did not perform more in-depth qualitative interviews to confirm this. However, from the results reported by by Tabori et al., we conclude that the PedsQL GI CF symptoms and concerns are important [8].

Compared to other gastro-intestinal diseases, patients with CF reported better GI QOL: data for Crohn’s disease, ulcerative colitis, functional abdominal pain, irritable bowel syndrome showed values for the total PedsQL GI <80% for all conditions [22–23,32–33]. However, the data for patients with CF are skewed, as a quarter of the patients has a result <80% and the median results for some of the subscores are comparable to other diseases. A possible explanation for the relatively high scores in patients with CF is that they are all treated with PERT and followed in specialized CF centres, resulting in a median, almost normal BMI which would suggest no significant residual malabsorption [34]. From another perspective, gastro-intestinal symptoms might not be considered very problematic by patients with CF who have several other problems (i.e. respiratory symptoms, nasal symptoms) which is not the case in purely gastro-intestinal diseases. This however contradicts recent patient survey results [29].

## Conclusion

In conclusion, important steps were made towards the validation of the existing PedsQL GI for use in children with CF and we are confident that the CF PedsQL GI, its shortened version, holds promise to be used both for clinical purposes and has potential as an outcome measure for clinical trials involving the gastro-intestinal aspects of CF in children. A next important step in the validation of the CF PedsQL GI is to show response to an intervention.

## Supporting information

S1 Table Overview of age-appropriate versions of all used questionnaires for patients and their parents: PedsQL GI, CFQ-R and VAS. Abbreviations: CFQ-R: Cystic Fibrosis Related Quality of Life score–Revised, IC: informed consent, PIF: patient information file, PedsQL GI: Pediatric Quality of Life Inventory, Gastrointestinal Symptoms Module, VAS: Visual Analogue Scale.
(DOCX)

S2 Table Results for total PedsQL GI and subscales for parents at baseline, compared to parents of healthy controls. Note that no data were available in literature for the subscales ‘Medicines’ and ‘Communication’. Subscales with a mean score below 95% in parents of patients with CF are marked in bold. Significant differences between parents from patients and those of healthy controls are marked with *.
(DOCX)

S3 Table Results for total PedsQL GI and subscales for subgroups of patients according to age group. Results for Cronbach’s alpha are also included in the table per age category. Knowing that the subgroups are smaller than the total group, these values are obviously lower in some subgroups. Results that indicate a ceiling effect for a certain score in any of the subgroups is marked in italics.
(DOCX)

S4 Table Results for total PedsQL GI and subscales for subgroups of parents according to age group of their children. Results for Cronbach’s alpha are also included in the table per age category. Knowing that the subgroups are smaller than the total group, these values are obviously lower in some subgroups. Results that indicate a ceiling effect for a certain score in any of the subgroups is marked in italics.
(DOCX)

S5 Table Results for correlations between total PedsQL GI and CF PedsQL GI and subscales of CFQ-R. This table shows the data for teens. Data for children and parents were similar (not shown).
(DOCX)

S6 Table Dataset used for statistical analysis. (XLSX)

S1 Fig Correlation between total PedsQL GI score and the CF PedsQL GI. CF PedsQL GI is the sum of the 9 selected and informative subscales.
(TIF)

## Abbreviations

APP: Mobile application
BMI: Body Mass Index
CDC: Centers for Disease Control and Prevention
CF: Cystic Fibrosis
CF PedsQL GI: Shortened, Cystic Fibrosis specific version of PedsQL GI
CFQ-R: Cystic Fibrosis Related Quality of Life score—Revised
CI: Confidence interval
FEF_25-75_: Forced expiratory flow between 25–75% of the FVC
FEV_1_: Forced expiratory volume in 1 second
FVC: Forced vital capacity
GI QOL: Gastro-intestinal related Quality of life
GLI: Global Lung Initiative
IC: Informed consent
ICC: Intra Class Correlation Coefficient
MID: Minimal important difference
PedsQL GI: Pediatric Quality of Life Inventory, Gastrointestinal Symptoms Module
PERT: Pancreatic Enzyme Replacement Therapy
PI: Pancreatic insufficiency
PIF: Patient information file
PRO: Patient Reported Outcome
QOL: Quality of Life
VAS: Visual Analogue Scale
