# Supplementary material for: Assessing gastro-intestinal related quality of life in cystic fibrosis: Validation of PedsQL GI in children and their parents
Source: PLoS One. 2019 Dec 20;14(12):e0225004. doi: 10.1371/journal.pone.0225004 (PMC6924691; doi:10.1371/journal.pone.0225004)
Supplement: S2 Table — Note that no data were available in literature for the subscales ‘Medicines’ and ‘Communication’. Subscales with a mean score below 95% in parents of patients with CF are marked in bold. Significant differences between parents from patients and those of healthy controls are marked with *. (DOCX) [file pone.0225004.s002.docx]

| **Variable** | **Parents of Healthy Controls^17^** | **Parents of patients with CF**  **n = 248** | **Cronbach’s**  **Alpha** | **Linear Regression** |
| --- | --- | --- | --- | --- |
|  | Median (1st, 3rd Q.) | Median (1st, 3rd Q.) | α | P-value |
|  |  |  |  | Difference [95% CI] |
| **Total PedsQL GI** | 90.19 (80.43, 99.72) | 85.3 (79.33, 91.6) | 0.94 | P = 0.002* |
|  |  |  |  | -3.35 [-5.43, -1.26] |
| **Stomach Pain** | 82.64 (66.5, 96.53) | 83.3 (67.73, 100) | 0.89 | P = 0.68 |
|  |  |  |  | 0.68 [-2.61, 3.96] |
| **Stomach Discomfort** | 91.45 (78.1, 100) | 95 (80, 100) | 0.84 | P = 0.12 |
|  |  |  |  | 2.36 [-0.59, 5.3] |
| Food and Drink Limits | 93.95 (80.71, 100) | 100 (87.5, 100) | 0.88 | P = 0.08 |
|  |  |  |  | 2.40 [-0.31, 5.12] |
| Trouble Swallowing | 98.65 (89.98, 100) | 100 (100, 100) | 0.71 | P = 0.44 |
|  |  |  |  | 0.74 [-1.14, 2.62] |
| **Heartburn and reflux** | 93.86 (84.62, 100) | 93.8 (81.2, 100) | 0.61 | P = 0.08 |
|  |  |  |  | -1.92 [-4.04, 0.21] |
| Nausea and vomiting | 93.86 (84.62, 100) | 100 (87.5, 100) | 0.92 | P = 0.89 |
|  |  |  |  | -0.17 [-2.65, 2.30] |
| **Gas and bloating** | 89.11 (73.52, 100) | 71.4 (57.1, 85.7) | 0.90 | P < 0.001* |
|  |  |  |  | -13.84 [-17.31, -10.37] |
| **Constipation** | 92.15 (79.46, 100) | 82.1 (71.85, 91.1) | 0.90 | P < 0.001* |
|  |  |  |  | -6.49 [-9.12, -3.87] |
| Blood in bowel movement | 98 (89.26, 100) | 100 (100, 100) | 0.76 | P < 0.001* |
|  |  |  |  | 4.51 [2.89, 6.13] |
| **Diarrhea** | 96.98 (86.65, 100) | 92.9 (78.6, 100) | 0.84 | P < 0.001* |
|  |  |  |  | -4.57 [-6.54, -2.61] |
| Worry about bowel movements | 97.69 (89.42, 100) | 100 (88.75, 100) | 0.76 | P = 0.17 |
|  |  |  |  | -1.43 [-3.46, 0.61] |
| **Worry about stomach aches** | 95.93 (82.24, 100) | 87.5 (75, 100) | 0.89 | P < 0.001* |
|  |  |  |  | -6.63 [-9.82, -3.43] |
| **Medicines** |  | 81.2 (68.8, 93.8) | 0.65 |  |
|  |  |  |  |  |
| **Communication** |  | 85 (60, 100) | 0.85 |  |
|  |  |  |  |  |
