# Supplementary material for: Assessing gastro-intestinal related quality of life in cystic fibrosis: Validation of PedsQL GI in children and their parents
Source: PLoS One. 2019 Dec 20;14(12):e0225004. doi: 10.1371/journal.pone.0225004 (PMC6924691; doi:10.1371/journal.pone.0225004)
Supplement: S4 Table — Results for Cronbach’s alpha are also included in the table per age category. Knowing that the subgroups are smaller than the total group, these values are obviously lower in some subgroups. Results that indicate a ceiling effect for a certain score in any of the subgroups is marked in italics. (DOCX) [file pone.0225004.s004.docx]

|  | | Parents of Children 2-4 years n=50 | | | Parents of children 5-7 years  n = 52 | | | Parents of children 8-12 years  n = 73 | | | Parents of children 13-18 years  n = 73 | |
| --- | --- | --- | --- | --- | --- | --- | --- | --- | --- | --- | --- | --- |
|  | Median (1st, 3rd Q.) | | Cronbach’s alpha | Median (1st, 3rd Q.) | | Cronbach’s alpha | Median (1st, 3rd Q.) | | Cronbach’s alpha | Median (1st, 3rd Q.) | | Cronbach’s alpha |
| Total PedsQL GI (%) | 89.5 (86.72, 92.3) | | 0.93 | 84.65 (75.22, 89.98) | | 0.92 | 82.6 (76.62, 90.67) | | 0.95 | 83.95 (79.23, 92.98) | | 0.95 |
| Stomach Pain (%) | 87.5 (75, 100) | | 0.87 | 81.25 (69.77, 95.8) | | 0.84 | 79.2 (65.65, 92.73) | | 0.92 | 79.2 (62.5, 100) | | 0.90 |
| Stomach Discomfort (%) | *95 (85, 100)* | | 0.84 | 95 (80, 100) | | 0.81 | 95 (80, 100) | | 0.84 | 95 (77.5, 100) | | 0.86 |
| Food Drink Limits (%) | *100 (91.7, 100)* | | 0.87 | *95.8 (81.25, 100)* | | 0.82 | *100 (85.4, 100)* | | 0.90 | *100 (87.5, 100)* | | 0.90 |
| Trouble Swallowing (%) | *100 (100, 100)* | | 0.58 | *100 (100, 100)* | | 0.75 | *100 (100, 100)* | | 0.68 | *100 (100, 100)* | | 0.77 |
| Heartburn reflux (%) | 93.8 (87.5, 100) | | 0.37 | 93.8 (81.2, 100) | | 0.52 | 93.8 (81.2, 100) | | 0.61 | 87.5 (75, 100) | | 0.69 |
| Nausea vomiting (%) | *100 (87.5, 100)* | | 0.95 | *100 (87.5, 100)* | | 0.93 | 100 (87.5, 100) | | 0.87 | *100 (81.2, 100)* | | 0.94 |
| Gas and bloating (%) | 76.8 (60.7, 90.2) | | 0.90 | 67.9 (57.1, 87.5) | | 0.89 | 71.4 (50, 82.1) | | 0.91 | 71.4 (57.1, 85.7) | | 0.89 |
| Constipation (%) | 85.7 (80.4, 87.5) | | 0.83 | 80.4 (67, 88.4) | | 0.91 | 83 (69.6, 92.9) | | 0.92 | 82.1 (75, 94.6) | | 0.91 |
| Blood bowel movement (%) | *100 (100, 100)* | | 0.75 | *100 (100, 100)* | | 0.81 | *100 (100, 100)* | | 0.59 | *100 (100, 100)* | | 0.89 |
| Diarrhea (%) | *96.4 (90.2, 100)* | | 0.85 | 85.7 (78.6, 96.4) | | 0.74 | 87.5 (75, 97.3) | | 0.77 | 92.9 (78.6, 100) | | 0.91 |
| Worry bowel movements (%) | *100 (95, 100)* | | 0.84 | *100 (82.5, 100)* | | 0.73 | 95 (90, 100) | | 0.76 | *97.5 (80, 100)* | | 0.74 |
| Worry stomach aches (%) | *100 (87.5, 100)* | | 0.82 | 87.5 (62.5, 100) | | 0.84 | 81.25 (75, 100) | | 0.92 | 75 (62.5, 100) | | 0.89 |
| Medicines (%) | 93.8 (81.2, 100) | | 0.71 | 87.5 (75, 95.35) | | 0.55 | 81.2 (68.8, 87.5) | | 0.60 | 75 (65.65, 93.8) | | 0.71 |
| Communication (%) | *100 (90, 100)* | | 0.92 | 85 (57.5, 100) | | 0.83 | 75 (60, 90) | | 0.84 | 77.5 (63.75, 100) | | 0.83 |
