# Supplementary material for: Assessing gastro-intestinal related quality of life in cystic fibrosis: Validation of PedsQL GI in children and their parents
Source: PLoS One. 2019 Dec 20;14(12):e0225004. doi: 10.1371/journal.pone.0225004 (PMC6924691; doi:10.1371/journal.pone.0225004)
Supplement: S5 Table — This table shows the data for teens. Data for children and parents were similar (not shown). (DOCX) [file pone.0225004.s005.docx]

| **Teenagers CFQR's Subscales** | **CF PedsQL GI**  *Spearman's rho* | **PedsQL GI**  *Spearman's rho* |
| --- | --- | --- |
| *Physical* | 0.343, p < 0.001 | 0.454, p < 0.001 |
| *Vitality* | 0.298, p = 0.002 | 0.431, p < 0.001 |
| *Emotion* | 0.225, p = 0.02 | 0.354, p < 0.001 |
| *Eating* | 0.314, p = 0.001 | 0.412, p < 0.001 |
| *Treatment Burden* | 0.287, p = 0.03 | 0.362, p = 0.002 |
| *Health Perceptions* | 0.288, p = 0.003 | 0.414, p < 0.001 |
| *Social* | 0.18, p = 0.07 | 0.268, p = 0.007 |
| *Body* | 0.328, p < 0.001 | 0.399, p < 0.001 |
| *Role* | 0.439, p < 0.001 | 0.542, p < 0.001 |
| *Weight* | 0.195, p = 0.051 | 0.300, p = 0.002 |
| *Respiratory* | 0.308, p = 0.002 | 0.411, p < 0.001 |
| *Digestion* | 0.564, p < 0.001 | 0.623, p < 0.001 |
