# Supplementary figures and images for: Assessing gastro-intestinal related quality of life in cystic fibrosis: Validation of PedsQL GI in children and their parents
Source: PLoS One. 2019 Dec 20;14(12):e0225004. doi: 10.1371/journal.pone.0225004 (PMC6924691; doi:10.1371/journal.pone.0225004)

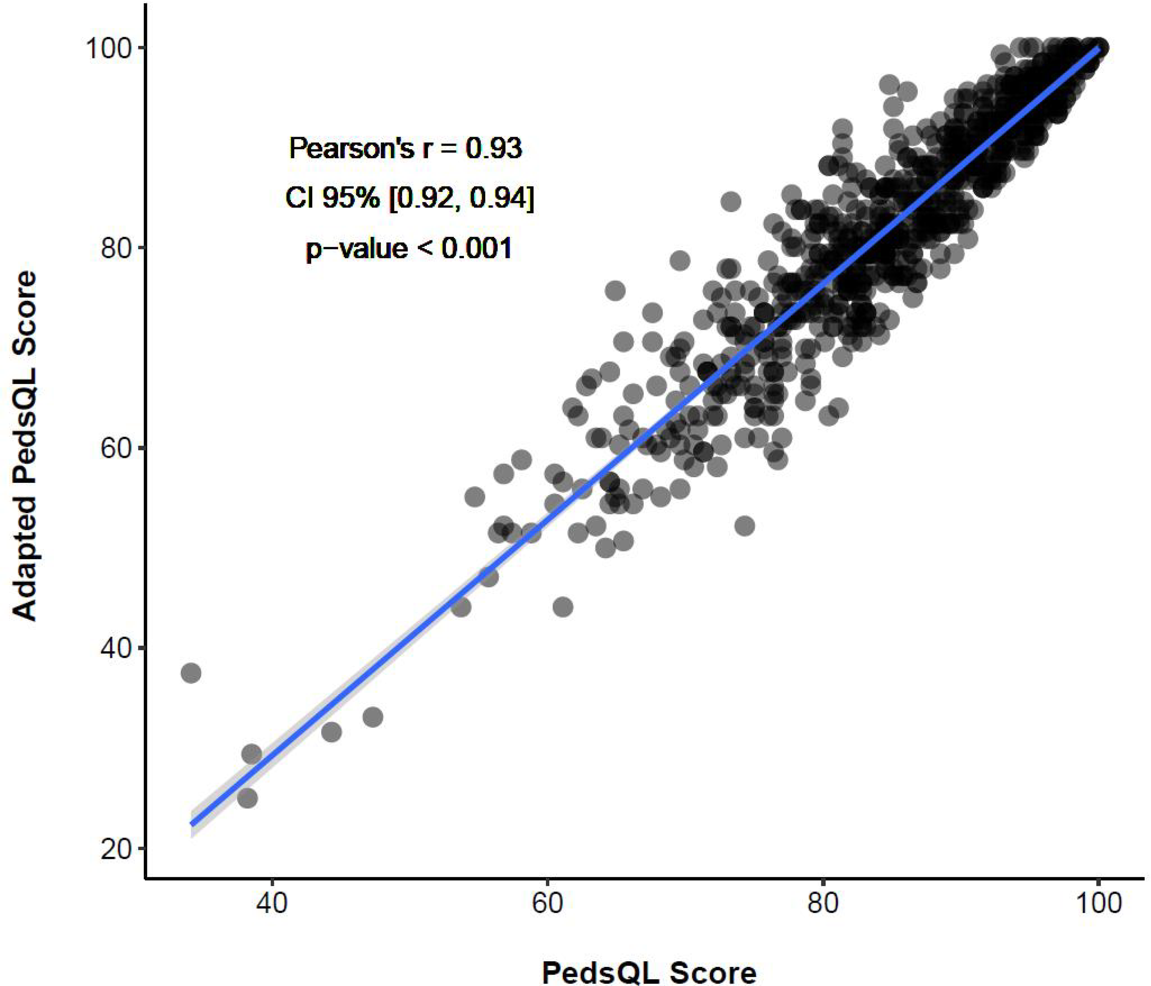

Supplement: S1 Fig — CF PedsQL GI is the sum of the 9 selected and informative subscales. (TIF) [file pone.0225004.s007.tif]
