# Supplementary material for: Assessing gastro-intestinal related quality of life in cystic fibrosis: Validation of PedsQL GI in children and their parents
Source: PLoS One. 2019 Dec 20;14(12):e0225004. doi: 10.1371/journal.pone.0225004 (PMC6924691; doi:10.1371/journal.pone.0225004)
Supplement: Supplementary file 7 [file pone.0225004.s001.docx]

	IC	***PIF***	***PedsQL GI***	***CFQ-r***	***VAS***
2-4 yrs	IC parent	PIF parent	Peds QL toddler 2-4: parent	/	VAS parent (about child)
5 yrs	IC parent	PIF parent	PedsQL GI young children 5-7: parent	/	VAS parent (about child)
			PedsQL GI young children 5-7: child (interviewer format smiley's)		
6 yrs	IC parent	PIF parent	PedsQL GI young children 5-7: parent	CFQ-r parent	VAS parent (about child)
			PedsQL GI young children 5-7: child (interviewer format smiley's)	CFQ-r child (interviewer format)	
7 yrs	IC parent	PIF parent	PedsQL GI young children 5-7: parent	CFQ-r parent	VAS parent (about child)
			PedsQL GI young children 5-7: child (interviewer format smiley's)	CFQ-r child (interviewer format)	VAS child
8-11 yrs	IC parent	PIF parent	PedsQL GI children 8-12: parent	CFQ-r parent	VAS parent (about child)
			PedsQL GI children 8-12: child (self report)	CFQ-r child (interviewer format)	VAS child
12 yrs	IC parent	PIF parent	PedsQL GI children 8-12: parent	CFQ-r parent	VAS parent (about child)
	Assent child	PIF child	PedsQL GI children 8-12: child (self report)	CFQ-r child (self report)	VAS child
13 yrs	IC parent	PIF parent	PedsQL GI teens 13-18: parent	CFQ-r parent	VAS parent (about child)
	Assent child	PIF child	PedsQL GI teens 13-18: child (self report)	CFQ-r child (self report)	VAS child
14-18 yrs	IC parent	PIF parent	PedsQL GI teens 13-18: parent	CFQ-r teen (self report)	VAS parent (about child)
	Assent child	PIF child	PedsQL GI teens 13-18: child (self report)		VAS child
